# Advancements in Predictive Tools for Primary Graft Dysfunction in Liver Transplantation: A Comprehensive Review

**DOI:** 10.3390/jcm13133762

**Published:** 2024-06-27

**Authors:** Piotr Gierej, Marcin Radziszewski, Wojciech Figiel, Michał Grąt

**Affiliations:** Department of General Transplant and Liver Surgery, Medical University of Warsaw, 02-091 Warsaw, Poland

**Keywords:** liver transplantation, graft dysfunction, primary nonfunction, retransplantation

## Abstract

**Highlights:**

**What are the main findings?**

- Primary graft dysfunction (PGD) involves early allograft dysfunction (EAD) and more severe primary nonfunction (PNF), both stemming from ischemia–reperfusion injury (IRI).
- Accurate and early diagnosis of PGD is crucial to the retransplantation decision-making process and the implementation of future mitigation strategies.
- Novel tools for predicting PNF utilize serum markers, marker-derived models, tissue biopsy analysis, metabolomics, evaluations of organ perfusion and liver metabolism, and assessments of graft perfusate during machine perfusion.

**What is the implication of the main finding?**

- The current implementation of extended criteria donors (ECD) exacerbates the limitations of the binary EAD criteria and has prompted a refreshed approach to the graft dysfunction assessment protocol.
- Recently, new serum parameters have been associated with EAD occurrence, and their incorporation into graft dysfunction evaluations may improve accuracy.

**Abstract:**

Orthotopic liver transplantation stands as the sole curative solution for end-stage liver disease. Nevertheless, the discrepancy between the demand and supply of grafts in transplant medicine greatly limits the success of this treatment. The increasing global shortage of organs necessitates the utilization of extended criteria donors (ECD) for liver transplantation, thereby increasing the risk of primary graft dysfunction (PGD). Primary graft dysfunction (PGD) encompasses early allograft dysfunction (EAD) and the more severe primary nonfunction (PNF), both of which stem from ischemia–reperfusion injury (IRI) and mitochondrial damage. Currently, the only effective treatment for PNF is secondary transplantation within the initial post-transplant week, and the occurrence of EAD suggests an elevated, albeit still uncertain, likelihood of retransplantation urgency. Nonetheless, the ongoing exploration of novel IRI mitigation strategies offers hope for future improvements in PGD outcomes. Establishing an intuitive and reliable tool to predict upcoming graft dysfunction is vital for early identification of high-risk patients and for making informed retransplantation decisions. Accurate diagnostics for PNF and EAD constitute essential initial steps in implementing future mitigation strategies. Recently, novel methods for PNF prediction have been developed, and several models for EAD assessments have been introduced. Here, we provide an overview of the currently scrutinized predictive tools for PNF and EAD evaluation strategies, accompanied by recommendations for future studies.

## 1. Introduction

The incongruity between the demand and supply of organs in transplant medicine has resulted in a mortality rate of approximately 20–25% among patients on the waiting list for liver transplantation, thus necessitating the use of extended criteria donors (ECD) [1]. To address the challenges of organ scarcity and to improve outcomes for patients awaiting hepatic transplantation, donors with features, such as macrovesicular steatosis, viral infections, or post circulatory death (DCD), have been incorporated into the donor pool. However, graft quality has emerged as a critical determinant of postoperative liver performance, and the likelihood of primary graft dysfunction (PGD) has been assessed as higher among recipients of ECD-derived livers [2].

Primary graft dysfunction (PGD) comprises early allograft dysfunction (EAD) and more severe primary nonfunction (PNF). Although there is no consensus regarding the definition of EAD, a widely accepted description was proposed by Olthoff et al. in 2010 to encompass at least one of the following during the first 7 days after transplantation: bilirubin concentration ≥ 10 mg/dL on day 7, INR ≥ 1.6 on day 7, AST or ALT activity > 2000 U/L during the initial 7 days [3]. The occurrence of EAD in post-transplant patients is associated with higher mortality rates and diminished graft survival. Primary nonfunction is typically defined as death or retransplantation within the first week after surgery, without other discernible causes (e.g., infection or graft rejection).

Although EAD and PNF describe different clinical outcomes, the underlying cause is believed to be similar and is attributed to both ischemia–reperfusion injury (IRI) and mitochondrial damage [4]. Retransplantation can often be avoided in the case of EAD, but it remains the only effective treatment for PNF. However, alternative mitigation strategies, such as inhaled nitric oxide or the administration of prostaglandins, are being actively explored [5]. Furthermore, the development of hypothermic oxygenated perfusion (HOPE) and normothermic regional perfusion (NRP) has improved graft quality in ECD and introduced a novel opportunity for organ assessment and the prediction of postoperative complications [6].

Establishing an intuitive and reliable tool to foresee upcoming graft dysfunction is crucial for early high-risk patient identification and an appropriate retransplantation decision-making process. Accurate diagnostics for PNF and EAD are essential initial steps for implementing future mitigation strategies. Recently, novel methods for PNF prediction have been developed, and several models for EAD assessment have been introduced. Here, we provide an overview of the currently scrutinized predictive tools for PNF and EAD evaluation strategies, accompanied by recommendations for future studies.

Several of the tools described in this article were evaluated based on their c-statistic performance. The c-statistic, also referred to as the concordance statistic or the area under the receiver operating characteristic curve (AUC-ROC), gauges the discriminatory power of a predictive model. In essence, it measures how effectively a model distinguishes between patients with a specific condition and those without a specific condition. A c-statistic value of 0.5 indicates no discriminatory power, while a value of 1.0 signifies perfect discrimination.

## 2. Ischemia–Reperfusion Injury (IRI) on the Cellular Level

Primary graft dysfunction (PGD) is associated with ischemia–reperfusion injury (IRI), which is caused by the disruption and subsequent restoration of blood supply during organ procurement and liver transplantation. A comprehensive grasp of the underlying mechanisms responsible for IRI is essential for the intentional development of diagnostic tools. Its complex cellular and molecular processes not only impact the liver itself but also result in systemic injury. Numerous participants, including hepatocytes, neutrophils, Kupffer cells, platelets, hepatic stellate cells (HSCs), and liver sinusoidal endothelial cells (LSECs), are involved in the occurrence of IRI [7].

**Figure 1:**
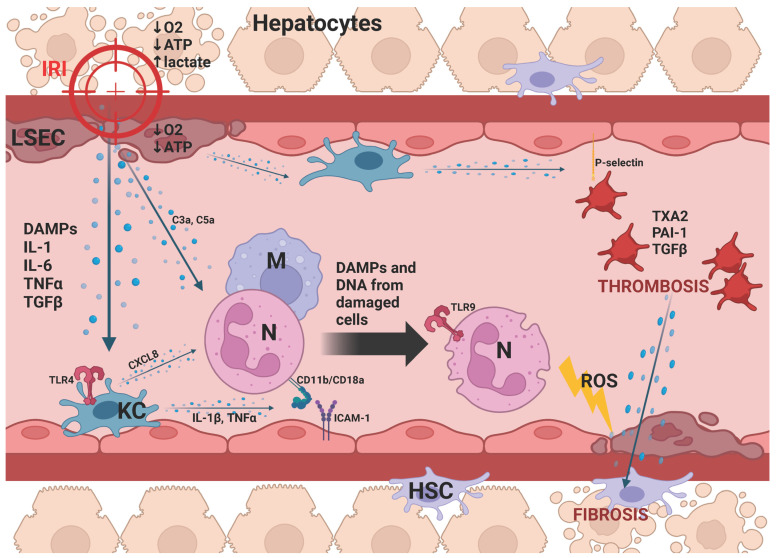
Illustrating the lumen of a liver sinusoid. Ischemia–reperfusion injury (IRI) damages hepatocytes and liver sinusoidal endothelial cells (LSECs). Ischemia and the lack of an oxygen supply disrupt mitochondria and adenosine triphosphate (ATP) production, resulting in impaired gluconeogenesis and lactate accumulation. Inflammatory cytokines released from apoptotic cells stimulate Kupffer cell (KC) inflammatory activation and trigger neutrophil (N) and macrophage (M) migration to the liver. Cytokines produced by Kupffer cells (KCs) increase the chemotaxis of neutrophils (N) and stimulate their adhesion to endothelial cells. Damage-associated molecular patterns (DAMPs) and DNA fragments derived from IRI-induced cell death trigger neutrophil (N) degranulation and reactive oxygen species (ROS) secretion, further damaging the liver tissue. At the same time, inflammatory cytokines increase P-selectin expression on the membrane of liver sinusoidal endothelial cells (LSECs), causing platelet aggregation. Activated platelets not only induce thrombosis in the transplanted graft but also release cytokines that stimulate hepatic stellate cells (HSCs) and initiate fibrotic changes. Abbreviations: ischemia–reperfusion injury (IRI), liver sinusoidal endothelial cell (LSEC), adenosine triphosphate (ATP), Kupffer cell (KC), neutrophil (N), macrophage (M), damage-associated molecular patterns (DAMPs), reactive oxygen species (ROS), hepatic stellate cell (HSC), interleukin 1 beta (IL-1β), interleukin 6 (IL-6), tumor necrosis factor alpha (TNFα), transforming growth factor beta (TGFβ), toll-like receptor (TLR), complement anaphylatoxins (C3a, C5a), intracellular adhesion molecule-1 (ICAM-1), integrin receptor (CD11b/CD18a), thromboxane 2A (TXA2), and plasminogen activator inhibitor-1 (PAI-1). Created with BioRender.com (accessed on 26 May 2024).

Termination of the initial blood supply decreases the amount of oxygen received by hepatocytes and liver sinusoidal endothelial cells (LSECs), leading to mitochondrial damage, ATP depletion, and increased lactate accumulation [8]. This results in IRI-induced hepatocyte and LSEC death accompanied by the release of inflammatory cytokines (IL-1β, IL-6, TNFα, TGFβ), damage-associated molecular patterns (DAMPs), and DNA fragments. Resident liver macrophages (Kupffer cells) stimulated by these factors increase toll-like receptor (TLR) expression and initiate chemokine 8 (CXCL8) secretion, which binds to complement proteins (C3a and C5a) derived from damaged LSECs and hepatocytes, and induces the migration of neutrophils to the liver as the organ reperfuses [9,10]. Kupffer cells also release IL-1β and TNFα, which promote adhesion between neutrophils (CD11b/CD18a) and LSECs (ICAM-1) [11]. The further neutrophil degranulation and reactive oxygen species (ROS) secretion, stimulated mainly by DAMPs and DNA from the damaged cells, exacerbate the destruction of liver tissue [12]. At the same time, increased P-selectin expression on the surface of the epithelium activates platelets, which release thromboxane 2A (TXA2), plasminogen activator inhibitor-1 (PAI-1), and TGFβ, leading to thrombosis and HSC-dependent graft fibrosis [13,14]. Figure 1 depicts the cellular mechanism behind the liver IRI. 

## 3. Prediction of Primary Nonfunction

### 3.1. Serum Lactate Concentration

**Figure 2:**
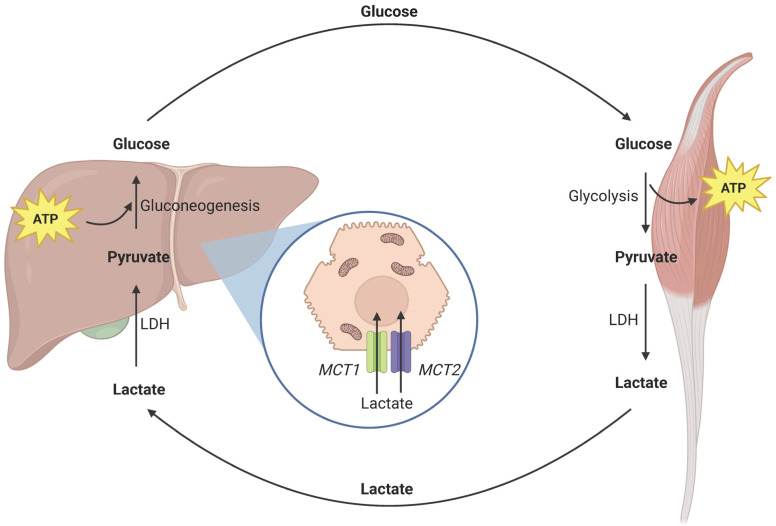
Illustration of the lactic acid cycle (Cori cycle). Lactates, produced mainly in the muscles, cannot be efficiently metabolized in the liver due to ischemia–reperfusion injury (IRI) and the lack of ATP in hepatocyte mitochondria. Hyperlactatemia is exacerbated by the failure of MCT1 and MCT2 membrane transporters, leading to dysfunctional lactate uptake by the hepatocyte. Abbreviations: adenosine triphosphate (ATP), lactate dehydrogenase (LDH), monocarboxylate transporter (MCT1, MCT2). Created with BioRender.com (accessed on 26 May 2024).

Ischemia and the impaired oxygen supplementation of hepatocytes result in insufficient ATP synthesis in mitochondria [7]. The lack of ATP, which is the main energy currency of the cell, prevents the metabolism of lactate within the hepatocyte. This inhibits the lactic acid cycle (also known as the Cori cycle) in which lactate, produced mainly in muscles, is converted to pyruvate and used for gluconeogenesis (Figure 2). This leads to hyperlactatemia, which is further increased by reperfusion injury, damaging the hepatocyte lactate transporters (MCT1, MCT2) and disrupting the uptake of lactates in the liver [15]. Therefore, the serum lactate concentration may correlate with the severity of IRI and indicate upcoming PGD. 

A practical application of this theory was recently described by Golse et al. [16], who observed that the intraoperative arterial lactate concentration shortly after liver reperfusion could serve as an early predictor of PGD. In their study on a group of 296 patients, a lactate concentration ≥ 5 mmol/L at the end of liver transplantation predicted PNF with a sensitivity of 83.3% and a specificity of 74.3%. Furthermore, the addition of lactate measurements to the BAR-score improved its predictive power regarding 3-month patient survival from an increase in the c-statistic of 0.74 to 0.87. 

On the other hand, Galli et al. [17], in a larger cohort of 1137 patients, obtained a sensitivity of 61% and specificity of 68% for PNF prediction when a cutoff of 5 mmol/L was applied and a sensitivity of 44% and specificity of 96% for arterial blood lactate ≥ 9.5 mmol/L. 

In another study, conducted in a pediatric liver transplantation group, higher lactate levels were also reported among children experiencing PNF [18]. Moreover, a postoperative serum lactate level > 3 mmol/L correlated with biliary complications and mortality, with an AUROC of 0.73 and 0.72, respectively, in a group of 145 patients. 

The lactate concentration is a promising prognostic tool for PNF prediction; however, further research and validation in large cohort studies are required. Its main advantages are simplicity, accessibility, and the possibility of early calculation. Furthermore, similar tools that rely on serum lactate levels, such as the lactate-to-platelet ratio and the lactate-to-venous-arterial CO_2_ and O_2_ ratio, have not yet been comprehensively assessed in terms of PNF diagnostics [19,20].

### 3.2. Scoring Models and Alternative Serum Markers

Inflammation and graft fibrosis resulting from IRI exert an omnidirectional systemic effect. PNF cases, such as those with severe IRI, should mirror these changes in laboratory findings associated with liver function. Al-Freah et al. [21] developed a PNF-predicting model based on a combination of the albumin concentration at the time of transplant surgery, lactate level and AST activity on posttransplant day 1, INR value and bilirubin concentration on day 3, and AST activity on day 7. They introduced the King’s College Hospital PNF score (King-PNF score) for a group of 1286 patients undergoing liver transplantation, of whom 3.7% were diagnosed with postoperative PNF. The proposed model achieved a c-statistic of 0.912 for the training cohort and 0.831 for the validation cohort, outperforming previously developed systems. However, PNF has been defined as death or retransplantation within the first two weeks following surgery, which deviates from the commonly proposed timeframe of one week. In an external validation conducted by Nie et al. [22], the King-PNF score predicted PNF occurrence with an AUROC of 0.891, superseding both the MEAF and lactate-adjusted BAR score (Table 1).

Although the King-PNF score remains superior to other models in terms of accuracy, its calculation still requires 7 postoperative days (3 days in the modified version), which is a major obstacle to early PNF diagnostics and is at a disadvantage when compared to arterial blood lactate measurements. Several recent studies have suggested that other parameters that may improve PNF prediction models include C-reactive protein (CRP), serum urea, and procalcitonin [23,24]. In addition, a study by Fukazawa et al. [25] showed that the predictive power of serum markers could be improved when adjusted for donor-recipient size mismatch. A novel target was proposed by Li et al. [26], who observed that levels of extracellular histones (released during IRI-induced cell death along with DNA and DAMPs) were significantly higher in liver recipients experiencing PGD during the first 72 h after surgery.

### 3.3. Assessment during Machine Perfusion

The development of hypothermic oxygenated perfusion (HOPE) and normothermic machine perfusion (NMP) has not only improved graft quality and reduced the risk of severe graft-related complications but also introduced novel opportunities for liver assessments prior to transplantation [27,28]. The graft perfusate can provide crucial information regarding organ viability, potentially identifying patients at a high risk for the occurrence of PNF. Various markers, including AST, ALT, CRP, lactate dehydrogenase (LDH), and hyaluronic acid (HA), have been proposed as prospective targets; however, none of them have been validated or introduced into routine clinical practice [29]. Nevertheless, they still represent a promising topic of interest for future analyses. 

Recent articles have advocated for routine liver assessments during ex situ hypothermic oxygenated perfusion to improve the decision-making process on whether to transplant the organ or not. Eden et al. [4] proposed flavin mononucleotide (FMN), nicotinamide adenine dinucleotide + hydrogen (NADH), and mitochondrial CO_2_ production during machine perfusion for screening mitochondrial functions prior to transplantation. Patrono et al. [30] emphasized that lactate clearance during NMP is a key prerequisite for graft acceptance, especially in low-quality organs. Verhelst et al. reported a very promising analysis of glycome patterns in the perfusate derived from hepatic venous flushing prior to transplantation. A single glycan, agalacto core-alpha-1,6-fucosylated biantennary glycan (NGA2F), was significantly elevated among patients suffering from graft dysfunction and was able to predict PNF with 100% accuracy in a study group of 122 patients [31]. 

A further investigation of injury markers during the organ-preservation phase and the development of a comprehensive model could enable effective PGD prediction during the pre-transplantation period. Therefore, highly damaged organs can be identified and rejected early, while moderate-risk graft recipients can be carefully monitored in order to implement mitigation strategies or secondary transplantation as early as possible.

### 3.4. Role of the Liver Biopsy

Another alternative strategy that can identify damaged organs is liver biopsy, thus potentially facilitating early PGD prediction. Among the various methods of tissue analysis, a growing interest has been observed in the use of metabolomic biosignatures for preoperative graft biomarker assessments. Cortes et al. [32] used mass spectrometry coupled with liquid chromatography to measure levels of phospholipids, lysophospholipids, sphingomyelins, bile acids, and histidine metabolism products, revealing impaired lipid and histidine pathways in dysfunctional grafts. This model can predict EAD preoperatively with 91% sensitivity and 82% specificity while accurately classifying all PNF cases into the graft dysfunction group. Recently, Zhang et al. [33] applied metabolomics to create the graft metabolite- and clinical parameter-based PNF (GMCP-PNF) predictive model. This innovative system achieved a c-statistic of 0.965 (100% sensitivity, 85% specificity) for preoperative PNF prediction by incorporating both clinical features (graft weight, warm and cold ischemia time, donor total bilirubin, AST, ALT, anhepatic time, and steatosis) and the metabolomic biosignature of eight key metabolites. Furthermore, they identified 59 disparately expressed metabolic features between EAD and PNF cases, suggesting potential differences in disease etiology between these conditions. In addition to metabolomics, electron microscopy can provide preoperative information about donor liver function by displaying ultrastructural changes indicative of hepatocyte injury [34]. 

Liver biopsy analysis can also provide crucial information regarding the probability of graft dysfunction when performed directly after organ reperfusion. Severe IRI observed on histopathological examination, including necrosis, steatosis, and monomorphonuclear infiltrates, indicates an elevated likelihood of PGD [35,36,37]. Bruns et al. [38] emphasized that microarray analysis and quantitative real-time PCR can be used for post-reperfusion tissue biopsy evaluation, reporting 20 markers that may be able to predict postoperative graft dysfunction. Khorsandi et al. [39] showed that the microRNA expression profile could shed more light on the prediction of PNF, with miR-22 being of utmost importance. Nevertheless, due to the small size of the study groups in both trials, further research is needed.

### 3.5. Measurement of Graft Metabolism and Perfusion

IRI-induced mitochondrial damage impairs the metabolic performance of the graft, while platelet aggregation disrupts blood flow and further inhibits metabolic pathways by reducing the oxygen supply. Therefore, measurement of the liver function, as well as graft perfusion, can assess the IRI and the increased risk of PNF occurrence. 

This chain of events, primarily metabolic activity, can be evaluated using the maximal enzymatic liver function capacity (LiMAx) test [40]. It measures the level of metabolites in exhaled air after 13C-methacetin administration, which is metabolized by hepatic cytochrome P-450. Lock et al. [41] applied this method to predict critical complications after liver transplantation in a group of 99 patients. Cutoff values of 64 µg/kg/h directly after surgery and 42 µg/kg/h on the first postoperative day (POD) achieved a sensitivity of 1.0 (0.31–1.0) and a specificity of 1.0 (0.94–1.0) for PNF prediction, outperforming the AST activity measurement. Nevertheless, due to the small size of the study group, this encouraging result requires further validation. 

Another approach that focuses particularly on the perfusion aspect of graft dysfunction is the indocyanine green fluorescence imaging (ICGFI) technique. Upon intravenous administration, indocyanine green (ICG) passes through the circulatory system bound to plasma proteins until it reaches the liver, where ICG is taken up by hepatocytes and secreted directly into the bile. A near-infrared camera detects fluorescence signals emitted by ICG; thus, the ICG fluorescence pattern can indicate hepatocyte disfunction, as well as impaired blood flow or bile secretion. 

Levesque et al. [42] measured the plasma disappearance rate (PDR) of ICG during the first 5 days following liver transplantation and found that a PDR of ICG lower than 12.85%/min was predictive of hepatic artery thrombosis, sepsis, and PNF. They also suggested that a sequential decline in PDR before POD5 might indicate graft acute rejection. A few years later, Olmedilla et al. [43] introduced a scoring system that relied on postoperative ICG clearance. Patients were divided into four categories ranging from 0 to 3 based on the PDR of ICG. The highest scoring group had a 50% risk of early death (within 30 days) or retransplantation (within 7 days), compared to a risk of 4.4%, 6.5%, and 12% for categories 0, 1, and 2, respectively. Recently, Figueroa et al. [44] created an intraoperative classification encompassing three types of ICG fluorescence, which incorporated ICG parenchymal uptake and the homogeneity of perfused areas. Their research showed that abnormal fluorescence patterns recorded immediately after organ reperfusion could identify patients at risk of PGD development. 

As an alternative to the ICG clearance, Narasaki et al. [45] recommended measuring the fluorescence intensity (FI) of the liver surface following ICG administration to evaluate its function. Although their study examined patients undergoing hepatopancreatobiliary surgery due to various types of carcinoma, they developed a mathematical model to describe the measured FI curves and calculated the parameters that define the temporal course of FI. Their findings provided the basis for a follow-up study by Dousse et al. [46], who expanded the previous model and introduced a novel parameter called “a_150_” in liver transplantation settings. This parameter reflects the speed at which the fluorescent signal reaches the plateau phase, and its value is negatively correlated with the time required for the signal to reach the plateau phase (the higher the a_150_ value, the shorter the time required, and the faster the plateau is achieved). Furthermore, its measurement was restricted to a predefined time period of 150 s. In their study, including 76 liver transplantations, an a_150_ value ≥ 0.0155 s^−1^ predicted 3-month graft survival with a sensitivity of 83.3% and a specificity of 78.6%, whereas, an a_150_ value ≥ 0.0178 s^−1^ diagnosed PNF with a sensitivity of 75% sensitivity and a specificity of 81.9%. Moreover, the authors reported that an arterial lactate concentration ≥ 5 mmol/L at the end of liver transplantation was a risk factor for the occurrence of PNF (OR = 13.15, *p* = 0.02) and suggested that the combination of a_150_ and lactate levels could be used for PNF prediction.

Overall, PDR and FI following ICG administration emerged as a promising tool for assessing postoperative liver function and the early prediction of PGD. However, the ICG pathway strongly depends on graft perfusion and bile production; therefore, its accuracy in PNF diagnostics may be significantly reduced by other conditions, such as hepatic artery thrombosis or biliary complications. In theory, this could limit the specificity of such tests as a predictive tool for PNF, as different outcomes could obtain results above the cutoff threshold. 

Nevertheless, disturbed blood flow stands as an important factor in graft failure development. A study by Matsushima et al. [47] conducted on a large study group of 1001 individuals showed that intraoperative portal flow below 65 mL/min/100 g (liver weight) predicted poorer 1-year graft survival and demonstrated an elevated likelihood of severe reperfusion injury, EAD, and PNF. On the other hand, patients with high portal flow (≥155 mL/min/100 g) were more often complicated by hepatic artery thrombosis and biliary disturbances; however, intraoperative hepatic flow was lower in this group. Recently, Zheng et al. [48] proposed that a hemodynamic graft assessment prior to the transplantation procedure relies on Doppler ultrasonography (DUS) and contrast-enhanced ultrasonography (CEUS) performed on donors prior to surgical procurement. They observed that reduced enhancement of donor livers on CEUS is a risk factor for PGD development; therefore, it can provide important information even before organ procurement. 

Examinations to assess graft perfusion expand the pool of potential predictive tools for PNF; however, they are unlikely to be able to diagnose PNF alone because of the many factors that influence their outcome, so combinations with some alternative methods may be beneficial. Perhaps the combination of a metabolic measurement and a graft perfusion assessment could provide a universal tool in the future. 

## 4. Assessment of Early Allograft Dysfunction

The primary objective of introducing early allograft dysfunction (EAD) was to develop a tool capable of defining the poor initial function of the transplanted organ, thereby providing an endpoint other than graft loss or the patient’s death. In addition, such a tool would be able to accurately assess which patients are at a high risk of postoperative graft failure, and thus, they require comprehensive postoperative monitoring and early intervention. Despite the unanimous agreement on the need for such parameters, there is no consensus regarding their definition. Currently, the most widely accepted EAD description is that proposed by Olthoff et al. [3] more than a decade ago. They defined EAD as the fulfilment of at least one of the following conditions within the first 7 days after transplantation: bilirubin concentration ≥ 10 mg/dL on day 7, INR ≥ 1.6 on day 7, and AST or ALT activity > 2000 U/L during the initial 7 days. Subsequent research has validated that EAD occurrence (as defined by the criteria of Olthoff et al.) in the posttransplant period is associated with inferior graft outcomes and worse survival rates [49]. 

Recently, due to global organ shortages, grafts obtained from extended criteria donors (ECD) have been incorporated into clinical practice, resulting in an elevated incidence of EAD [50,51]. However, despite the higher likelihood of EAD in ECD-derived graft recipients, they are not associated with poorer graft or patient survival [50,51,52]. However, this information is inconclusive, as other researchers have observed increased complication rates among ECD recipients [53]. This may be due to the nonhomogeneous definitions of ECD across different articles.

Nonetheless, this issue highlights the major shortcomings of the current EAD definition. While patients undergoing EAD experience elevated rates of mortality and morbidity, the rigid and binary nature of EAD complicates the prediction of postoperative courses. Many patients who meet the EAD criteria achieve long-term survival without significant complications, whereas those who do not fall into the EAD category may encounter postoperative sequelae. Consequently, some authors have advocated for reconsidering the definition of early graft dysfunction by proposing a more flexible and progressive formulation. The overarching goal of this revised approach is to accurately evaluate the risk of graft failure, particularly with regard to early graft failure (EGF), which is usually described as graft loss or the patient’s death within the first 3 months following transplantation. Table 2 provides an overview of alternative models for the assessment of early graft dysfunction that have been developed over the last 10 years. They are compared in terms of their c-statistic for the prediction of EGF. A brief description of the models discussed in this article is shown in the Supplementary Table S1. 

The intricacies of predicting postoperative graft failure were already highlighted in 2013 by Wagener et al. [54], who emphasized the limited accuracy and predictive power of the EAD construct. Consequently, they explored alternative solutions, revealing that the Model for End-Stage Liver Disease (MELD), which is widely used in pre-transplant graft allocation, can be used as a predictive tool for early graft loss and mortality when applied on the 5th postoperative day. A follow-up study by Benko [55] supported these findings by achieving an even higher c-statistic by using a MELD score cutoff of 16, compared to 18.9 in the previous article. However, both studies were conducted on relatively small groups of patients (572 and 116, respectively), necessitating further validation. Meanwhile, Angelico et al. [56] introduced the Donor-Recipient Allocation Model (DReAM) in a much larger study group, surpassing some previously developed scoring systems. The c-statistic obtained by DReAM was comparatively modest; however, in the subsequent validation group of 448 patients, this value had improved to 0.76. 

In 2015, Pareja et al. [57] described the Model for Early Allograft Function scoring (MEAF) and its potential to predict graft loss or a patient’s death within 90 days of liver transplantation. It is also noteworthy that the likelihood of PNF was significantly elevated with an MEAF score higher than 7. A further study by Jochmans et al. [58] demonstrated that MEAF predicted EGF with a c-statistic of 0.727 (rising to 0.782 in a multivariable model) and outperformed the binary EAD model (AUROC of 0.644). Moreover, Richards et al. [59] highlighted the superior sensitivity of MEAF, as it can be calculated earlier than EAD. Furthermore, a recent external validation by Nie et al. [22] showed that MEAF predicted PNF and EGF with a c-statistic of 0.872 and 0.802, respectively, in a group of 720 patients. 

Another model introduced in 2018 by Agopian et al. [60] is the Liver Graft Assessment Following Transplantation (L-GrAFT). In a study group of 2008 patients, there was a better prediction of 90-day graft loss or mortality by AUROC than by the MEAF and EAD models. Further multicenter validation, conducted by the same research team, showed c-statistics of 0.78, 0.72, and 0.68 for L-GrAFT, MEAF, and EAD, respectively, in a study group comprising 3201 patients, as well as 0.81, 0.57, and 0.64 in a study group of 171 participants [61]. 

One of the most recent models for the assessment of postoperative graft dysfunction was developed by Avolio et al. [62] for the Early Allograft Failure Simplified Estimation (EASE) score. The c-index of this model was 0.87 and 0.78 in two study groups, 1609 and 538, respectively, outperforming both L-GrAFT and MEAF scores. 

Other systems for assessing graft dysfunction developed in the last decade include the ABC model introduced by Rhu et al. [63], as well as the system proposed by Diaz-Nieto et al. [64]. The Diaz-Nieto model relies on postoperative serum transaminase activity and stratifies patients into four groups with a different risk of 30-day graft failure. The strengths of this system include the possibility of early evaluation and a relatively straightforward calculation process. Nevertheless, both models either lack external validation or behave similarly to the binary EAD model. 

Overall, most newly developed systems are more accurate than binary EAD, even when adjusted for the graft-to-recipient weight ratio [65]. The most impressive c-statistic among the graft dysfunction assessment models was achieved with the Comprehensive Complication Index (CCI), predicting 90-day graft loss with an AUROC of 0.94; however, the c-statistic in the validation group was significantly lower, yielding 0.77 [66]. Nevertheless, contemporary literature posits that the L-GrAFT and EASE scores are emerging as the most auspicious indicators for EGF prognostication [67]. The dynamic MEAF model outperformed the binary EAD definition in terms of EGF prediction accuracy and took less time to calculate; however, MEAF achieved a lower c-statistic in almost every study compared to the EASE and L-GrAFT models. Interestingly, the King-PNF score also performed excellently in EGF prediction [22]. Moreover, MELD calculated based on POD5 is a promising tool as it obtains a high c-statistic; however, an external validation with a large study group is required. Another advantage of the postoperative MELD calculation is that it has been widely used and recognized worldwide.

**Table 2:** Overview of models developed over the past 10 years to assess postoperative graft dysfunction. These systems were compared in terms of their predictive power for early graft failure (EGF), which is understood to be patient death or graft loss within the first 90 days following surgery.

Year	Study [Ref.]	Size	Outcome	Incidence	Model	c-Statistic
2013	Wagener et al. [54]	572	EGF	7%	MELD	0.81
2014	Angelico et al. [56]	2864	EGF	7%	BAR	0.57
					D-MELD	0.58
					SOFT	0.59
					DReAM	0.66
2015	Pareja et al. [57]	829	EGF	10%	MEAF	no data
2017	Benko et al. [55]	116	EGF	10%	MELD	0.84
2017	Jochmans et al. [58]	660	EGF	12%	MEAF	0.73
					EAD	0.64
2018	Agopian et al. [60]	2008	EGF	11%	L-GrAFT	0.83
					MEAF	0.70
					EAD	0.68
2019	Diaz-Nieto et al. [64]	1194	Graft failure within 30 days	9%	Diaz-Nieto score	no data
2020	Richards et al. [59]	183	Graft failure within 28 days	8%	MEAF	0.74
2020	Avolio et al. [62]	1609	EGF	7%	EASE	0.87
					DRI	no data
					EAD	0.72
					D-MELD	0.70
					ET-DRI	0.63
					MEAF	no data
					L-GrAFT	0.85
		538		8%	EASE	0.78
					DRI	0.57
					EAD	0.63
					D-MELD	0.72
					ET-DRI	0.58
					MEAF	0.73
					L-GrAFT	0.71
2021	Lai et al. [66]	1262	EGF	15%	CCI	0.94
					MELD	0.60
					D-MELD	0.60
					BAR	0.60
					EAD	0.58
		520		3%	CCI	0.77
					MELD	0.57
					D-MELD	0.57
					BAR	0.56
					EAD	0.47
2021	Agopian et al. [61]	3201	EGF	7%	L-GrAFT	0.78
					MEAF	0.72
					EAD	0.68
		171		4%	L-GrAFT	0.81
					MEAF	0.57
					EAD	0.64
2021	Rhu et al. [63]	1153	Graft failure within 2 months	7%	ABC Model	0.73
					MEAF	0.69
					EAD	0.66
		359		13%	ABC Model	0.74
					MEAF	0.71
					EAD	0.66
2022	Manzia et al. [65]	331	EGF	16%	mEAD	0.74
					EAD	0.64
		123		5%	mEAD	0.68
					EAD	0.52
2023	Moosburner [67]	906	EGF	no data	DRI	0.50
					ET-DRI	0.54
					D-MELD	0.59
					MEAF	0.72
					L-GrAFT	0.80
					EASE	0.80
					ABC Model	0.68
					EAD	0.69
					BAR	0.60
2023	Nie et al. [22]	720	EGF	9%	MEAF	0.80
					King-PNF score	0.87
					BAR-Lac	0.76

### Alternative Assessment of Allograft Dysfunction

On the other hand, most of the currently available EAD assessment models rely on overlapping parameters; however, they vary in terms of formula calculations. Meanwhile, an ongoing study has revealed novel laboratory markers associated with EAD occurrence. Implementing some of these findings into the EAD assessment model may improve its accuracy. Therefore, in Table 3, we provide an overview of serum parameters that have been reported to be associated with EAD development over the past 10 years. Furthermore, if any of these markers were validated in a large external study while maintaining a high discriminatory power, it would supersede pre-existing EAD assessment models due to the lower complexity and earlier calculation. These parameters also represent a promising spectrum of targets for further PNF prediction research. The majority of these markers are derived from current knowledge regarding the IRI mechanism at the cellular level.

Nunez et al. [68] observed that an elevated serum level of the complement anaphylatoxins C3a and C5a correlated with graft steatosis and EAD occurrence; however, it did not achieve a significant effect. They also noted that interleukin-33 (IL-33) is one of the damage-associated molecular patterns (DAMPs) released by IRI-injured hepatic cells, and its elevation corresponds to graft dysfunction [69]. A further study by Barbier et al. [70] demonstrated an increase in the concentration of IL-33 in patients undergoing EAD. In a group of 40 patients, IL-33 possessed a c-statistic value of 0.76 for predicting graft dysfunction. The role of IL-33 in the IRI mechanism is currently under scrutiny. In addition to serving as a DAMP, recent studies have shown that IL-33, which is mainly released from LSECs, stimulates inflammation by increasing neutrophil extracellular trap formation while possessing a protective effect on hepatocytes [71,72].

In addition, the interleukin-6 (IL-6) concentration may possess a predictive value as it is also released from damaged liver cells and plays a significant role in inflammation signaling. Faitot et al. [73] reported that the IL-6 level at the time of reperfusion correlates with graft survival. However, some older articles describe its limited potential to predict postoperative outcomes; therefore, further research is needed [74,75]. Interestingly, Chae et al. [76] observed an elevated EAD incidence among patients with increased preoperative IL-6.

Karakhanova et al. [77] noted that the preoperative concentration of interferon-gamma (IFNɣ) correlated with postoperative life expectancy and predicted EAD with an AUROC of 0.76. They also observed that EAD cases expressed higher levels of interleukin-10 (IL-10) and chemokine-10 (CXCL10) within the initial postoperative days and that the c-statistic for IL-10 on EAD prognostication amounted to 0.84. These findings are particularly interesting because it is currently believed that inflammatory cytokines and DAMPs, released by IRI-affected apoptotic cells, activate Kupffer cells to produce an inflammatory phenotype and inhibit the production of immunosuppressive IL-10 [7]. Perhaps its increased concentration is derived from regulatory T cells, which secrete IL-10 in response to IFNɣ released by Kupffer cells [7,77,78]. Furthermore, Karakhanova et al. [76], in contrast to Chae et al. [77], did not observe a preoperative change in IL-6 between the EAD and non-EAD groups.

A recent study by Faria et al. [79] showed that elevated high-mobility group box 1 protein (HMGB1) and nucleosomes belonging to DAMPs correlated with EAD development and were significantly higher in PNF cases; however, due to the small size of the study group, this could be a random effect [79]. This corresponds to a study by Sosa et al. [80], which showed that the HMGB1 concentration correlated with the severity of IRI.

In addition, the increase in macrophage and neutrophil activity after reperfusion may reveal more clearly the severity of IRI. In a study group of 1960 participants, Kwon et al. [81] observed that EAD patients had increased neutrophil levels compared to the lymphocyte number. A neutrophil-to-lymphocyte ratio (NLR) ≥ 2.85 had a sensitivity of 64% and a specificity of 70% for EAD diagnostics. Thomsen et al. [82] noted that macrophage involvement can be measured based on soluble macrophage activation marker (sCD163) levels, which are elevated in EAD patients. Park et al. [83] proposed an increased C-reactive-protein-to-albumin ratio (CRP/ALB) as an EAD marker, as it should reflect ongoing inflammatory changes.

As an alternative to the immune response observation, the analysis of lipid metabolism is a very promising approach to assess graft dysfunction. Ceglarek et al. [84] investigated circulating liver metabolites of the cholesterol pathway, amino acids, and acylcarnitines in the plasma of graft recipients. By applying liquid chromatography/tandem mass spectrometry (LC-MS/MS), they observed that EAD cases were associated with lower circulating esterified sterol concentrations. The usefulness of metabolomics in liver transplantation was subsequently supported by Tsai et al. [85], who achieved an excellent c-statistic of 0.95 for EAD prediction by combining levels of cholesterol oleate, phosphatidylcholine (PC), and lysophosphatidylcholine (LysoPC). A few years later, the same research team found that the combination of betaine, palmitic acid, PC, and LysoPC could differentiate between EAD and non-EAD cases with an AUROC of 0.82, which was further increased to 0.85 by incorporating total bilirubin [86]. Furthermore, Yang et al. [87] observed that serum total cholesterol (sTC) below 1.42 mmol/L on POD3 correlated with a higher EAD incidence. These results indicate the potential of metabolomics in graft dysfunction evaluations; however, due to the small size of the study groups, these findings require further validation.

A study by Pollara et al. [88] showed that preoperative EAD prediction could be predicted by deceased donor plasma mitochondrial DNA (mtDNA) level measurements. The elevated mtDNA serum concentration is suspected to correlate with posttransplant complications because it may serve as a DAMP and amplify the immune response in graft recipients. Thus, it may simultaneously play a causative role and may be released from cells damaged by IRI. Further research by Yoshino et al. [89] noted elevated circulating mtDNA levels in graft recipients undergoing EAD.

Other parameters recently associated with EAD occurrence include phosphorus, B-type natriuretic peptide (BNP), and myoglobin (Mb), which are increased in EAD cases [90,91,92]. In a study by Chae et al. [91], a serum BNP level ≥ 100 pg/mL predicted EAD with an AUROC of 0.75, whereas Mb obtained a c-statistic of 0.66 in a study by Zhang et al. [92]. Gorgen et al. [93] observed a decrease in serum factor V on POD1 in EAD cases, whereas Nedel et al. [94] reported lower thrombin-activatable fibrinolysis inhibitor (TAFI) among patients suffering from graft dysfunction.

Overall, recent studies have expanded the spectrum of parameters associated with EAD occurrence and may improve the graft dysfunction assessment protocol. Some of them (e.g., serum analysis using metabolomics) have achieved excellent accuracy in EAD prediction, whereas others (e.g., mtDNA measurement) can provide crucial information even before organ procurement. However, the majority of these studies have been conducted in small study groups, so further research is needed. Nevertheless, if some of these markers, such as lipid pathway metabolites, confirm their accuracy in large-scale studies, they will significantly improve graft viability assessments. Moreover, some of the parameters may enhance the currently used EAD assessment models, of which the performance is still very limited.

**Table 3:** Overview of serum markers reported to be associated with EAD development over the past 10 years. ↑—increased concentration, ↓—decreased concentration.

Year	Study [Ref.]	Size	Outcome	Incidence	Markers
2013	Hong et al. [90]	304	EAD	16%	↑ phosphorus
2015	Nedel et al. [94]	21	EAD	10%	↓ TAFI
2016	Karakhanova et al. [77]	41	EAD	49%	↓ IFNɣ, ↑ IL-10, ↑ CXCL10
2016	Chae et al. [91]	104	EAD	30%	↑ BNP
2016	Ceglarek et al. [84]	40	MEAF ≥ 6.1	no data	↓ SIE%/↓ CHE%
2017	Yang et al. [87]	231	EAD	17%	↓ sTC
2018	Chae et al. [76]	226	EAD	12%	↑ IL-6, ↑ IL-17
2018	Faitot et al. [73]	274	EAD	29%	↑ IL-6
2018	Pollara et al. [88]	65	EAD	no data	↑ mtDNA
2019	Gorgen et al. [93]	227	EAD	27%	↓ factor V
2018	Tsai et al. [85]	51	EAD	24%	↓ cholesterol oleate, ↓ LysoPC, ↑ PC
2019	Kwon et al. [81]	1960	EAD	11%	↑ NLR
2019	Thomsen et al. [82]	27	EAD	59%	↑ sCD163
2019	Park et al. [83]	588	EAD	14%	↑ CRP/ALB
2020	Faria et al. [79]	22	EAD	50%	↑ HMGB1, ↑ nucleosome
2020	Nunez et al. [68]	99	EAD	no data	↑ IL-33, ↑ C3a, C5a
2021	Tsai et al. [86]	74	EAD	30%	↑ betaine, ↓ LysoPC, ↓ PC, ↑ palmitic acid
2021	Yoshino et al. [89]	21	EAD	33%	↑ cmtDNA
2021	Barbier et al. [70]	40	EAD	no data	↑ IL-33
2022	Zhang et al. [92]	150	EAD	35%	↑ Mb

Abbreviations: thrombin-activatable fibrinolysis inhibitor (TAFI), interferon-gamma (IFNɣ), interleukin (IL), chemokine (CXCL), B-type natriuretic peptide (BNP), esterified β-sitosterol (SIE), esterified cholesterol (CHE), serum total cholesterol (sTC), mitochondrial DNA (mtDNA), lysophosphatidylcholine (LysoPC), phosphatidylcholine (PC), neutrophil-to-lymphocyte ratio (NLR), soluble macrophage activation marker (sCD163), C-reactive protein to albumin ratio (CRP/ALB), high-mobility group box 1 protein (HMGB1), complement anaphylatoxins (C3a, C5a), circulation mitochondrial DNA (cmtDNA), myoglobin (Mb).

## 5. Conclusions and Recommendations

Despite the significant advances that have been made in the last decade, there is still no universal tool for primary nonfunction (PNF) prediction. Among the predictive models, the KING-PNF score seems to be the most accurate; however, the time required for its calculation limits its usefulness. The measurement of arterial blood lactate concentrations shortly after reperfusion offers a promising alternative due to its accessibility and the possibility of early evaluation, although current knowledge of its predictive power is inconclusive. In terms of accuracy, metabolomics and liver function capacity tests are the most encouraging solutions; nevertheless, further validation in large study groups is needed. Moreover, in the context of the increased utilization of machine perfusion, preoperative graft viability assessment emerges as a promising diagnostic window. Organ function evaluation could be improved by incorporating novel markers that have recently been associated with early allograft dysfunction (EAD) but have not yet been described in terms of PNF prediction. Furthermore, these parameters could improve currently available EAD-assessment models. Cytokine profiling has emerged as a prospective addition, and serum metabolomics also provide an excellent c-statistic for EAD prediction. Despite growing evidence of the need for more accurate and dynamic EAD assessment models, there is no unanimously accepted grading system. The inconclusiveness between outcome-defining studies hampers the evaluation of introduced models and markers, thus making it impossible to perform a reliable meta-analysis. Another factor exacerbating the analysis is the ongoing development of new systems without a thorough examination of some previous ones. Nonetheless, contemporary literature recognizes the EASE and L-GrAFT scores as the most accurate methods for early graft dysfunction assessments, and the MEAF remains a more efficient tool than the binary EAD definition. Furthermore, the incorporation of some novel serum markers may significantly improve their outcomes. This review summarizes current knowledge regarding primary graft dysfunction (PGD) prediction and lays the foundation for future studies on PNF prediction and EAD assessment improvements.

## Figures and Tables

**Table 1:** Comparison of the King’s College Hospital PNF score (King-PNF score) with other available models for predicting PNF.

Year	Study [Ref.]	Size	Outcome	Incidence	Model	c-Statistic
2017	Al-Freah et al. [21]	1125	PNF	3.70%	King-PNF score	0.91
					UK EGD Criteria	0.67
					US PNF Criteria	0.78
2023	Nie et al. [22]	720	PNF	3.90%	MEAF	0.87
					King-PNF score	0.89
					BAR-Lac	0.83
			EAD	39%	MEAF	0.84
					King-PNF score	0.81
					BAR-Lac	0.63
			early graft failure	9.30%	MEAF	0.80
					King-PNF score	0.87
					BAR-Lac	0.76

## Supplementary Materials

The following supporting information can be downloaded at: https://www.mdpi.com/article/10.3390/jcm13133762/s1, Table S1: A brief description of the graft dysfunction assessment models discussed in this article.
