# Supplementary material for: Advancements in Predictive Tools for Primary Graft Dysfunction in Liver Transplantation: A Comprehensive Review
Source: J Clin Med. 2024 Jun 27;13(13):3762. doi: 10.3390/jcm13133762 (PMC11242128; doi:10.3390/jcm13133762)
Supplement: Supplementary file 1 [file jcm-13-03762-s001.zip › jcm-3052520-supplementary.pdf]

Supplementary Table S1. A brief description of the graft dysfunction assessment models discussed in this article.

Name of the model	Abbreviation	Parameters required for calculation
Model for End-Stage Liver Disease	MELD	serum bilirubin, serum creatinine, international normalized ratio (INR)
Donor age adjusted MELD	D-MELD	donor age, preoperative MELD score
Delta MELD	delta MELD	maximum change in MELD score calculated at 2 time points between listing and transplantation
Early Allograft Dysfunction model	EAD	serum bilirubin, INR, aspartate aminotransferase (AST) or alanine aminotransferase (ALT) activity
Modified Early Allograft Dysfunction model	mEAD	serum bilirubin, INR, AST or ALT activity, graft-to-recipient weight ratio (GRWR)
Balance of Risk	BAR	recipient MELD score, cold ischemia time, reipient age, donor age, previous liver transplant (LT), life support dependence prior to transplant
Balance of Risk and Lactates	BAR-Lac	recipient MELD score, cold ischemia time, reipient age, donor age, previous liver transplant (LT), life support dependence prior to transplant, arterial blood lactate level at the end of liver transplantation
Donor Risk Index	DRI	donor age, race, height, cause and type of death, split/partial graft
Eurotransplant Donor Risk Index	ET-DRI	DRI, serum gamma-glutamyltransferase (GGT), rescue allocation (after at least three declines of “patient-oriented” organ offers due to poor organ quality the organ can be offered as a “centeroriented” offer to all recipients of a center)
Survival Outcomes Following Liver Transplant	SOFT	DONOR: age, cause of death, serum creatinine, type of procurement, cold ischemia time. RECIPIENT: age, BMI, prior transplants, previous abdominal surgeries, albumin level, pretransplant dialysis, type of pretransplant stay (home/ICU, hospitalized), MELD score, pretransplant life support, encephalopathy, portal vein thrombosis, portal bleed within 48h pretransplant, pretransplant ascites

Preallocation Survival Outcomes Following Liver Transplantation	P-SOFT	RECIPIENT: age, BMI, prior transplants, previous abdominal surgeries, albumin level, pretransplant dialysis, type of pretransplant stay (home/ICU, hospitalized), MELD score, pretransplant life support, encephalopathy, portal vein thrombosis, portal bleed within 48h pretransplant, pretransplant ascites
Donor-Recipient Allocation Model	DReAM	DONOR: age, cause of death, height, BMI, cold ischemia time. RECIPIENT: serum creatinine, serum bilirubin, aetiology of liver disease, prior upper abdominal surgery, portal vein thrombosis
Diaz-Nieto score	Diaz-Nieto score	recipient AST and ALT activity
Comprehensive Complication Index	CCI	postoperative complications
Model for Early Allograft Function Scoring	MEAF	recipient ALT, INR, serum bilirubin
Liver Graft Assessment Following Transplantation	L-GrAFT	recipient AST, INR, serum bilirubin, platelets count
Early Allograft Failure Simplified Estimation	EASE	recipient AST, platelets count, serum bilirubin, MELD score, packed red blood cell transfusions at LT, thrombosis of hepatic vessels, transplant center volume
ABC model	ABC	recipient AST, serum bilirubin, INR
Gala-Lopez score	Gala-Lopez score	DRI, recipient AST, ALT, serum bilirubin
King's College Hospital PNF score	King-PNF score	recipient AST, lactate level, serum bilirubin, INR, transplant albumin
NHS Blood and Transplant Liver Advisory Group criteria for super-urgent listing for LT for early graft dysfunction	UK PNF criteria	recipient AST, INR, lactate level, absence of bile production
Organ Procurement and Transplant Network urgent listing criteria for PNF	US PNF criteria	recipient AST, INR, acidosis (arterial pH≤7.30 or venous pH 7.25 and/or lactate≥4 mmol/L), anhepatic candidate
